# Reduction of a Strangulated Hernia, Apparently Effected by Acupuncture

**Published:** 1842-03

**Authors:** 


*Reduction of a Strangulated Hernia, apparently effected by Acupuncture.* By Dr. Daser.—A man, 50 years of age, was seized with strangulated hernia, with vomiting of stercoral matter and all the other usual symptoms. The taxis having failed, Dr. Daser, before having recourse to the operation for strangulated hernia, with the view of trying the effect of acupuncture, for the purpose of evacuting the gaseous contents of the strangulated portion of the intestine, made two punctures in it with a long fine needle. No gaseous matters apparently escaped, but the patient complained of acute pain, and loud gurgling sounds were heard in the abdomen, immediately after which the hernia was spontaneously reduced. Dr. Daser attributed this fortunate occurrence to the prick of the needle having excited contractibility of the intestine, causing it to contract on its contents, expel the gaseous matter into the abdominal portions of the gut, and thus facilitate its reduction.—*Ibid*, from *Journal fur Chirurgie und Augenhetlkunke,* 1840.

